# The Treatment of Insomnia of Pulmonary Tuberculosis

**Published:** 1902-10

**Authors:** S. C. Reisman

**Affiliations:** New York City; Assistant in the Out-Patient Department of Bellevue Hospital, New York, 220 East 78th Street.


### The Treatment of Insomnia of Pulmonary Tuberculosis.

By S. C. REISMAN, M.D., New York City.

Assistant in the Out-Patient Department of Bellevue Hospital, New York.

ONE of the most difficult and troublesome conditions to control effectively in pulmonary tuberculosis is the insomnia which so frequently accompanies this disease. A tubercular patient when he does not enjoy the recuperative influence of refreshing sleep undergoes all the tortures of Dante's condemned spirits.

This insomnia is due to various causes; either to excessive cough from the accumulation of secretions in the bronchial tubes, the irritating night-sweats, pleuritic pains, or to mere nervous excitability.

The effect of loss of sleep in these patients is to drag them nearer the approaching end. They become despondent, lose all hope (and the sufferer of this malady when he is able to sleep is, as we know, hopeful to the very last breath); they lose their appetite; the eyes bulge out of their sockets, and acquire a wild stare as in one demented—a group of symptoms which I can instantly recognise as a result of a loss of that "chief nourisher in life's feast," sleep.

Knowing the causes of the insomnia, the rational method of treatment is the employment of means to eliminate these disturbing factors. We are, however, but rarely able to accomplish this, and we are forced to treat the condition symptomatically; to resort to some measure that will produce undisturbed sleep, and shortly after its administration.

As it has been my fortune to treat a large number of patients suffering from this dread disease, and being dissatisfied with the numerous deleterious by-effects of the hypnotics of the pharmacopeia, I thought it worth while to investigate one of the newer sleep-producing remedies, named hedonal.

Professor Dreser, in his painstaking report on hedonal, explicitly states that the drug has absolutely no injurious effects on the heart, circulation and body temperature, and that it is completely oxidised in the system into water and carbonic acid, and hence I felt convinced that there was no danger of an accumulation of the drug in the body. I was further assured by several medical friends who had already employed this remedy that they had observed no toxic symptoms during its use, and though still distrustful I commenced to employ this remedy.

As a result of my careful investigation of hedonal I can most heartily confirm the opinions of its most enthusiastic admirers. The remedy has proven most valuable to me in the class of patients referred to. I have found it to be rapid and reliable in its action, non-irritating to the gastrointestinal, circulatory, or genitourinary systems, and devoid of after-effects. It has pronounced advantages over the older hypnotics, being superior to both morphin and chloral hydrate. It is better than morphin in two respects: first, it does not engender any craving; second, it does not produce any of the after-effects of morphin—namely, headache, nausea, disagreeable taste in the mouth and constipation. Over chloral hydrate hedonal has manifold advantages. It produces calm, refreshing sleep, even if the patient is suffering with pain; it creates no drug habit; it causes no gastric irritation, and at the same time it allays the harrassing bronchial sensitiveness.

I believe that hedonal will also be found valuable as a sedative in nervous diseases of a functional type and in conditions of cerebral congestion and irritation. I have not as yet, however, had sufficient opportunity to test it to my satisfaction, but I feel warranted in at least hinting at its expanded field of usefulness. I should be pleased to hear from my colleagues who have had experience with the use of hedonal in such diseases.

The following cases will serve to illustrate the mode of action of hedonal:

Case I.—F., a laborer, 31 years of age, a native of Poland; mother and sister both died of tuberculosis. He had been under treatment for three months, and complains of tingling in the throat, night sweats, moderate cough. His appetite is good and he is able to eat everything. Examination revealed crackling râles and broncho-vesicular breathing over both apices. Diagnosis: pulmonary tuberculosis. Under the administration of creosote he had been doing well. One week ago he was seized with a severe cough paroxysm. This kept him awake for three consecutive nights. It was a pitiful sight to see the remarkable change for the worse when he entered the dispensary. His eyes were bulging, glistening and staring, with dark rings around them. His appetite was gone, and he longed for death to release him from his troubles. I prescribed a powder of 30 grains of hedonal for the relief of the insomnia, and asked him to call at my office the next morning. The change at this time was striking. He had had a good night's rest, sleeping for 14 hours. During this time he awoke but once, falling quickly asleep again. He told me that his cough was quieter. I gave him 15 grains of hedonal and 1-24 grain heroin hydrochloride for the following night. This dose was not sufficient to produce the desired result. The next day he received 30 grains, which acted satisfactorily. The case is still under observation.

Case II.—J. G., a blacksmith, aged 43, has advanced tuberculosis of the left lung. His chief complaint was loss of sleep. He had a fair appetite, only slight cough, and moderate night-sweats. I gave him 15 grains hedonal, which secured a good night's rest. On the second night no effect was obtained from the 15 grain dose, which was increased to 25 grains. Sleep was produced, and the patient felt better in consequence. He has been receiving creosote all the time.

Case III.—R. B., trained nurse, 22 years of age; family history good; father and mother both living and in good health; brothers and sisters are well. The patient had been in good health till six months ago. For three months before this she had been in attendance upon a woman in the last stages of pulmonary tuberculosis. She apparently acquired the disease from the inhalation of germ-laden air, her strength being undermined. I found roughened breathing and dullness over both apices; temperature, 100° F.; pulse, 110; breathing rapid and shallow. Her body was poorly nourished and the lips cyanotic. She complained particularly of her inability to sleep at night, because her mind would not be quiet. I gave her 20 grains of hedonal and some creosote pills. After taking hedonal she enjoyed restful sleep with no after-effects.

Case IV., M. H., female, aged 47, complained of great weakness; had not had a good night's sleep for a month. She says she is very weak; has no appetite, and can barely walk. On examination I found a large cavity in the right lung. I prescribed 30 grains of hedonal and for the cough 1-24 grain of heroin hydrochloride. She returned in two days and reported feeling somewhat better, having slept after taking the powder for twelve hours, interrupted once by coughing. Hedonal was continued one week with no bad effects and an improvement of appetite.

Case V.—M. C., child, aged 11 years—an old tubercular case—complains of restlessness and sleeplessness. The cough was bad. After 15 grains of hedonal she slept soundly for six hours. The same dose repeated the next night afforded a sleep of eight hours' duration.

I have tried hedonal in eighteen tubercular patients and not in one instance has it failed to produce the desired effect. Of course, in many instances, I had to add heroin hydrochloride, 1-24 grain, to the hedonal in order to first quiet the cough.

The description of other cases would be but a repetition of the excellent sleep-producing properties of hedonal. I hesitated long before using the drug, as most physicians do with new remedies, but after my experience I can heartily and confidently recommend its trial. I believe it is destined to replace in many instances the established remedies where we desire a pure hypnotic free from all possibility of injurious action.

220 EAST 78TH STREET.

